# FCLQC: fast and concurrent lossless quality scores compressor

**DOI:** 10.1186/s12859-021-04516-7

**Published:** 2021-12-20

**Authors:** Minhyeok Cho, Albert No

**Affiliations:** Department of Electronic and Electrical Engineering, Hongik University, Seoul, Republic of Korea

**Keywords:** Concurrency, FASTQ, Lossless compressor, Quality score, Random access

## Abstract

**Background:**

Advances in sequencing technology have drastically reduced sequencing costs. As a result, the amount of sequencing data increases explosively. Since FASTQ files (standard sequencing data formats) are huge, there is a need for efficient compression of FASTQ files, especially quality scores. Several quality scores compression algorithms are recently proposed, mainly focused on lossy compression to boost the compression rate further. However, for clinical applications and archiving purposes, lossy compression cannot replace lossless compression. One of the main challenges for lossless compression is time complexity, where it takes thousands of seconds to compress a 1 GB file. Also, there are desired features for compression algorithms, such as random access. Therefore, there is a need for a fast lossless compressor with a reasonable compression rate and random access functionality.

**Results:**

This paper proposes a Fast and Concurrent Lossless Quality scores Compressor (FCLQC) that supports random access and achieves a lower running time based on concurrent programming. Experimental results reveal that FCLQC is significantly faster than the baseline compressors on compression and decompression at the expense of compression ratio. Compared to LCQS (baseline quality score compression algorithm), FCLQC shows at least 31x compression speed improvement in all settings, where a performance degradation in compression ratio is up to 13.58% (8.26% on average). Compared to general-purpose compressors (such as 7-zip), FCLQC shows 3x faster compression speed while having better compression ratios, at least 2.08% (4.69% on average). Moreover, the speed of random access decompression also outperforms the others. The concurrency of FCLQC is implemented using Rust; the performance gain increases near-linearly with the number of threads.

**Conclusion:**

The superiority of compression and decompression speed makes FCLQC a practical lossless quality score compressor candidate for speed-sensitive applications of DNA sequencing data. FCLQC is available at https://github.com/Minhyeok01/FCLQC and is freely available for non-commercial usage.

## Background

Since the Human Genome Project (HGP), sequencing technology has developed rapidly [1]. Recently proposed Next Generation Sequencing (NGS) technologies support massive parallel sequencing, which lowers sequencing costs. As a result, the amount of sequencing data increases dramatically. In 2025, it is expected that one Zettabase of new sequencing data will be generated every year [2]. The sequencing data is mainly stored in FASTQ format, which is widely being used in bioinformatics. The size of the FASTQ file is gigantic, where the size of human genome data ranges from tens to hundreds of gigabytes. For example, the size of the homo sapiens FASTQ file *SRR13587127* obtained from the Illumina HiSeq machine is 111 GB.

There exists a significant amount of recent works on FASTQ compression, including Spring [3], LFastqC [4], FQSqueezer [5], and fqzcomp [6]. Since the reads are sub-strings of the whole genome, there is much redundancy to be exploited for compression. Thus, recent works mainly focused on read compression. On the other hand, the quality scores have less statistical structure, and it is more challenging to compress [6]. Moreover, the quality scores occupy around 70% of losslessly compressed FASTQ file [7]. Thus, we need to focus on quality score compression.

Recently, several quality scores compression algorithms have been proposed, including qvz [8], crumble [9], MPEG-G [10], where the above works mainly considered lossy compression to boost the compression rate further. However, it is highly nontrivial to distinguish the critical component of the data, especially in medical applications, and therefore lossless compression is preferred [11]. Also, lossless compression is necessary for archiving purposes [12]. There are number of lossless quality scores compressors such as AQUa [13] and LCQS [14]. The above algorithms outperform the general-purpose compressors (such as Gzip), but the run time is significantly higher.

In this paper, we aim to design a fast lossless quality scores compressor. We propose Fast and Concurrent Lossless Quality scores Compressor (FCLQC) that achieves a comparable compression rate while having much faster than the baseline algorithms. We use concurrent programming to achieve fast compression and decompression. Concurrent programming executes a program independently, not necessarily simultaneously [15], which is different from error-prone parallel computing. We implement FCLQC using the modern language Rust [16].

### Why Rust?

Memory safety is essential for thread safety [17], and secure coding. While memory safety issue occurs in C and C++ code, most recent programming languages guarantee memory safety, Especially, Rust [16] supports the ownership and type systems that help manage memory safely and convert concurrency problems to compile-time errors.

Among many memory-safe programming languages, Rust is already gaining popularity. Rust was the second-fastest-growing language on the code-sharing platform in 2019 [18], and it has been the “most-loved” language for the last five years in a row according to Stack Overflow Developer Survey 2020 [19]. Similar to the Python Package Index (PyPI) for Python language, Rust also has *crates* which contains third-party packages for developers. It has more than 65,000 available packages, including a library of algorithms in bioinformatics [20]. It shows that there is not much overhead to use Rust instead of C++.

Our goal is to provide a thread-safe code that can handle more than 100 threads. Our experimental results contain a compression and decompression with 120 threads, which is proof of memory-safe code.

### FASTQ format

FASTQ file is a widely used data format that contains the output of sequenced data [21]. It has information of lots of genome fragments (called “read”). Each read information consists of four lines: (1) id (header), (2) read (nucleotides), (3) additional header, and (4) a line of quality values (also called quality scores). A line of quality values is a sequence of *Phred scores* $S=-10\log _{10} P$, where *P* corresponds to an estimate of the error probability of each nucleotide. Quality values are often stored in ASCII character of $Q=S+33$ (or $Q=S+64$), ranging from 33 to 73 (or from 64 to 104). Since id is short and nucleotides have four (A, C, G, T) possibilities, quality values are the most challenging components to compress [6]. The proposed algorithm focuses on compressing the quality values of a FASTQ file.

## Implementation

### Data modeling

Since there are various sequencing technologies from different entities including Illumina [22], OxfordNanopore [23], PacBio [24], and IonTorrent [25], we model the lines of quality values under minimal benign assumptions.

Suppose a FASTQ file consists of *N* reads of the same length *L*. Then, we have *N* lines of quality scores $\mathbf{Q }^{(1)}, \mathbf{Q }^{(2)}, \dots , \mathbf{Q }^{(N)}$, where each line of quality scores has length *L*, i.e., $\mathbf{Q }^{(i)} = (Q^{(i)}_1, \dots , Q^{(i)}_L)$. Since the quality scores tend to decrease within the line [8], we assume that the line of quality scores is a first-order Markov process. More precisely, the probability of the line of quality scores $\mathbf{Q }= (Q_1, \dots , Q_L)$ is given by

$$\begin{aligned} P(\mathbf{Q })&= P(Q_1, Q_2, \ldots , Q_L)\\&= P_m (Q_1) \prod _{j=2}^L P_c (Q_j|Q_{j-1}) \end{aligned}$$

for some marginal distribution $P_m(\cdot )$ and conditional distribution $P_c(\cdot |\cdot )$.

We further assume that the lines of quality scores are independent to each other, i.e., for $i\ne j$,

$$\begin{aligned} P(\mathbf{Q }^{(i)}, \mathbf{Q }^{(j)}) = P(\mathbf{Q }^{(i)}) P(\mathbf{Q }^{(j)}). \end{aligned}$$

Under the independence assumption, we compress the lines of quality scores separately, which allows concurrent programming. Our modeling is universal because we do not rely on any assumptions, including the range of quality scores, prior distributions, or length of quality scores.

### Algorithm overview

Although FCLQC is a compression algorithm for the quality scores, it can take the whole FASTQ file with id and read information as an input. In the first step (Split and Extraction), we extract the quality scores from the FASTQ file and divide them into multiple sub-files so that each thread can take care of the corresponding sub-file. Then, each thread computes the local statistics of its sub-file, where the main thread collects local statistics to estimate the probability distributions $P_m$ and $P_c$. In the second step (Compression), all thread’s estimated distributions are shared, and each thread compresses its sub-file with an arithmetic encoder. Details of “Split and Extraction” and “Compression” are provided below.

### Split and extraction

The file must be divided into sub-files for concurrent programming. The proposed scheme requires an input parameter $N_s$ at program execution. The *Splitter* splits the entire file into multiple sub-files, each containing $N_s$ lines of quality scores, i.e., $(\mathbf{Q }^{(1)}, \mathbf{Q }^{(2)}, \dots , \mathbf{Q }^{(N_s)})$. The number of sub-files is $\lceil \frac{N}{N_s}\rceil$.

To compress Markov source effectively, we need to estimate conditional and marginal probabilities. In our implementation, the *Counter* estimates marginal distribution ${\hat{P}}_m$ and a conditional distribution ${\hat{P}}_c$ by counting occurrences. We implement the *Counter* concurrently so that each thread has its *Counter* to extract the local statistics of quality scores from sub-files. These local statistics are merged into single summary statistics that represent the entire FASTQ file. The algorithm also stores the summary statistics since the decompressor also needs the marginal and conditional distributions. Note that each thread may use the local statistics from the corresponding sub-file; however, storing local statistics is also burdensome. Our experiment shows that the gain in compression rate using local statistics is not significant, and therefore we use summary statistics for simplicity.

### Compression

**Fig. 1:**
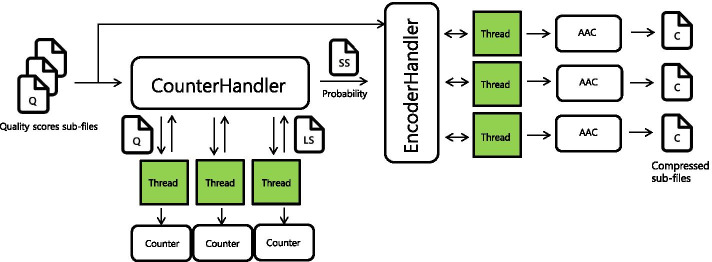
The general workflow of FCLQC after *Splitter*. *ConunterHandler* assigns sub-files (Q) to threads, and each thread counts the number of occurrences (LS) of quality scores in each file. The main thread aggregates all local count information (LS) and then generates summary statistics (SS) which contains estimated marginal and conditional distributions. The estimated distributions are passed to the *EncoderHandler*, and the *EncoderHandler* provides a sub-file with estimated distributions to each thread. Finally, each thread compresses quality scores of the divided file line by line using the adaptive arithmetic coder (AAC), and outputs a compressed sub-file (C)

The next step is an actual compression with an arithmetic encoder. More precisely, we implement an adaptive and concurrent arithmetic encoder for the first-order Markov process. The main thread assigns tasks to threads, and each thread concurrently encodes sub-files using its dedicated adaptive arithmetic encoder with summary statistics. The concurrent program allows the faster threads to compress more sub-files, while slower threads compress fewer sub-files. Our implementation is based on Rust standard concurrency library. Figure 1 shows a brief overview of our algorithm.

The decoding process also supports concurrency. In the decoding phase, each thread has its dedicated decompressor with summary statistics. The thread takes a compressed sub-file as an input and applies an arithmetic decoder to recover the original sub-file. Finally, we can recover the original quality scores by merging all decompressed sub-files.

Our algorithm supports random access, which refers to the ability to access a random location. Random access is convenient for many applications because it is inefficient to decompress the entire file to use a portion of the file. Note that outputs of an adaptive arithmetic encoder may have different numbers of bits for quality scores lines, which is usually a bottleneck for random access. To support random access, we generate a header for each line of quality scores that indicates the number of bits per line. The decompressor can find the exact location of the specific compressed quality scores by collecting header information.

In high-speed data compression, one of the challenges is a data writing step to a file. To minimize the I/O issue, we use the bit-buffers while compressing and decompressing the quality scores. For example, while compression, the buffer collects multiple compressed quality scores and flushes them to the output file.

## Results

This section describes the experimental results of proposed lossless quality scores compressor FCLQC as well as experimental setups. We have compared the performance of FCLQC with other baseline algorithms, including lossless quality scores compressor LCQS [14] and general-purpose compressors. All algorithms are tested with the recommended options that achieve the best compression rate.

We run experiments on Linux (Ubuntu LTS 20.04.2) with the following hardware specifications: AMD Ryzen Threadripper 3990X 64-Core Processor 128-thread and 128 GB of memory. The number of threads is a tunable parameter in FCLQC; however, we do not use all 128 threads because some algorithms do not have an option to adjust the number of threads. We set the number of quality score lines $N_s$ for each sub-file accordingly so that the number of sub-files is 120. We discuss more the number of threads in the following sections.

### Datasets

For a fair comparison, we selected the same FASTQ files from the experiment in LCQS [14]. In addition, we experiment with the additional dataset from different species, file size, coverage, and sequencing technology to avoid a data-dependent bias. We also apply compression algorithms to the synthetic data (*syn_read1*, *syn_read2*) generated by SimNGS [26], which is not considered in [14]. The synthetic dataset is publicly available in *Synthetic, Mouse, and Sampled Human data (SMaSH)* [27]. Details of datasets are provided in Table 1.

**Table 1:** Details of quality scores datasets

Filename	Organism	Technology	Length	Size (MB)	Coverage
*SRR554369_1*	P.Aeruginosa	Illumina GAIIx	100	160	50x
*SRR554369_2*	P.Aeruginosa	Illumina GAIIx	100	160	50x
*SRR327342_1*	S.Cerevisiae	Illumina GAII	63	918	175x
*SRR327342_2*	S.Cerevisiae	Illumina GAII	75	1090	175x
*SRR870667_1*	T.Cacao	Illumina GAIIx	108	7197	35x
*SRR870667_2*	T.Cacao	Illumina GAIIx	74	4952	35x
*syn_read1*	Synthetic	SimNGS	101	43,775	30x
*syn_read2*	Synthetic	SimNGS	101	43,775	30x

FCLQC has a preprocessing step that extracts quality scores from the FASTQ file. However, most other quality score compression algorithms and general-purpose algorithms cannot handle the raw FASTQ file. In the experiment, we extract quality scores from the FASTQ file, where all compression algorithms take extracted quality scores as an input. Also, we compute the compression ratio based on the file size of extracted quality scores.

### Baseline compressors

State-of-the-art FASTQ compression algorithms such as Spring [3], LFastqC [4], FQSqueezer [5], and fqzcomp [6] are focused on compressing the entire FASTQ file, especially the reads, rather than specializing in quality scores. Since the final compressed file of these algorithms includes ids and reads, it is hard to measure the compression rate of quality scores separately. For this reason, in this paper, we mainly consider the recently proposed lossless quality scores compressor LCQS [14], which can compress quality scores exclusively. LCQS optimized the compression ratio with robust quality score partitioning and adopted SIMD-based parallelization to boost compression speed. We believe that LCQS is a good baseline algorithm since only a few specialized compressors support random access and parallelization. Also, LCQS showed the best compression ratio and improved (de)compression speed for most datasets. Since LCQS has no parameters to tune, we apply LCQS in default mode.

Another quality score compressor AQUA [13] is considered. AQUa used multiple coding tools (such as different coder, average different coder, convolution predictors, etc.) with context-adaptive binary arithmetic coding (CABAC) scheme. We use the same parameters of AQUa that are described in [14]. Note that LCQS is complied with standard C++11 and g++ complier, while AQUa is implemented in JAVA.

Also, we consider general-purpose compressors, including 7-zip and pigz (parallelizable Gzip) widely used in practice. For pigz and 7-zip, we compress the quality score in the best compression mode in all experiments.

Each baseline algorithm supports different features, which are summarized in Table 2. Also, the details on the algorithm configurations are given in Table 3.

**Table 2:** Supported features of compressors

	FCLQC	LCQS	AQUA	7-zip	pigz
Without preprocessing	$\checkmark$	✕	✕	✕	✕
Random access	$\checkmark$	$\checkmark$	$\checkmark$	✕	✕
Multi-threading	$\checkmark$	$\checkmark$	✕	$\checkmark$	$\checkmark$
Custom number of threads	$\checkmark$	✕	✕	$\checkmark$	$\checkmark$

**Table 3:** Configurations for compressors

Compressor	Parameters	Source URL
FCLQC	Precision = 35 thead_num = 6 or 16	https://github.com/Minhyeok01/FCLQC
LCQS		https://github.com/SCUT-CCNL/LCQS
AQUa	Windowsize = 1, cabacgrouping=10485760	https://github.com/tparidae/AQUa
7-zip	-mx9(best) -mmt6 or -mmt16	https://www.7-zip.org/
pigz	-9(best) -p 6 or -p 16	https://zlib.net/pigz/

### Comparision: speed

The compression (or decompression) speed (MB/s) is measured by the ratio between the original file size (MB) of extracted quality scores and the compression time (seconds). Our experimental equipment has 64 cores and can use a maximum of 128 threads. However, we cannot manually adjust the number of threads of LCQS or AQUa. LCQS automatically adjusts the number of threads based on the file size. LCQS uses six threads when it compresses *SRR554369* while using 16 threads for other datasets. For a fair comparison, we limit the number of threads by 16 while testing FCLQC. Note that AQUa does not natively support multi-threading, so we measured compression speed by dividing the file.

Recall that FCLQC does not require a preprocessing of FASTQ files since it takes a whole FASTQ file as an input and divides the file into id, read, and quality scores. Because other baseline algorithms take a quality scores file (which can be viewed as a preprocessed file) as an input, we ignore the splitting time while measuring the running time of FCLQC.^1^

In Table 4, the compression speed and average memory usage of LCQS, AQUa, 7-zip, and pigz are presented. FCLQC shows an average compression speed of 137 (MB/s) when using six threads and 306 (MB/s) when using 16 threads, which is far better than the other baseline compressors on all datasets. Compared to LCQS, the performance gain is 31x to 46x. It provides more than 23x performance improvement over AQUa, 47x performance improvement over 7-zip, and 3x over pigz when using 16 threads. FCLQC also used less memory after pigz. LCQS requires more memory to compress small file sizes and considerably more memory, even for large files. On the other hand, FCLQC uses less memory compared to the file size. Thus, we can say that FCLQC performs better even in memory-constrained hardware environments.

**Table 4:** Comparison results of compression speed and average memory usage

Filename	Compression speed (MB/s)					Average memory usage (GB)				
	FCLQC	LCQS	AQUa	7-zip	Pigz	FCLQC	LCQS	AQUa	7-zip	Pigz
*SRR554369_1*	**135.59**	3.09	3.29	1.02	17.40	0.0132	1.76	0.59	0.63	**0.0126**
*SRR554369_2*	**139.13**	2.98	3.28	0.97	14.41	0.0132	1.38	0.58	0.63	**0.0126**
*SRR327342_1*	**305.64**	7.12	6.43	2.87	50.70	0.0134	7.68	0.60	3.52	**0.0126**
*SRR327342_2*	**301.67**	6.37	8.30	3.45	77.64	0.0133	7.93	0.59	4.41	**0.0126**
*SRR870667_1*	**341.74**	10.36	9.47	6.12	36.33	0.0133	10.71	0.62	7.43	**0.0126**
*SRR870667_2*	**316.58**	7.73	8.85	4.21	43.10	0.0134	9.57	0.61	7.56	**0.0126**
*syn_read1*	**292.03**	8.94	12.17	6.10	41.98	0.0134	14.24	0.62	12.12	**0.0126**
*syn_read2*	**275.52**	7.95	11.46	5.58	38.32	0.0133	14.31	0.61	12.04	**0.0126**

Bold denotes the fastest compression speed or lowest memory usage

Table 5 presents compression time with various numbers of threads while compressing *SRR870667_1* dataset. Compression time is measured only with 7-zip and pigz, which can adjust the number of threads. It is clear that FCLQC is the fastest in all the number of threads. Note that 7-zip cannot handle all threads properly when the number of available threads is more than 40. On the other hand, FCLQC can properly control all threads and compress the quality scores quickly.

**Table 5:** Compression time with the number of thread and CPU usage

Number of threads	Compression time (s)			Average CPU Usage (%)		
	FCLQC	7-zip	Pigz	FCLQC	7-zip	Pigz
1	**311.66**	9728.62	3080.33	100	100	100
10	**32.47**	1749.51	314.78	1000	600	1000
20	**21.37**	903.39	159.89	1900	900	2000
40	**7.37**	695.92	83.70	3200	2800	4000
60	**6.54**	480.06	60.13	5900	2800	6000
80	**5.91**	481.98	49.13	7300	2800	8000
100	**5.29**	478.78	42.41	9500	2800	10,000
120	**5.06**	481.53	36.63	11,600	2800	12,000

Bold denotes the lowest compression time

**Fig. 2:**
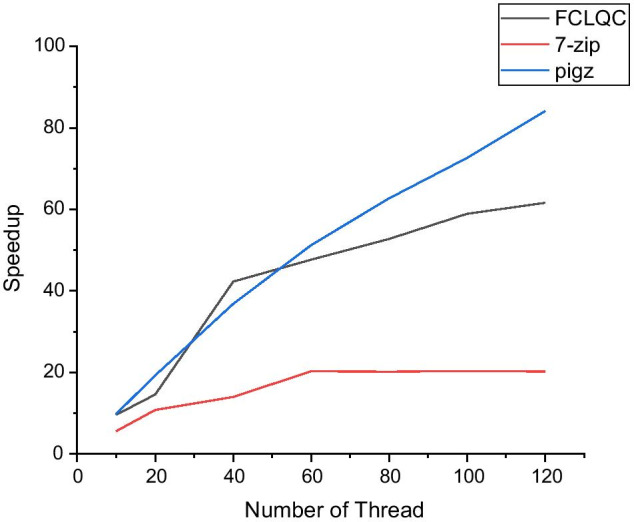
Speedup of FCLQC, 7-zip, and pigz where thread counts are from 10 to 120

Figure 2 shows speedup, the ratio between the single thread execution time and the parallel execution time. It is clear that 7-zip cannot handle more than 40 threads. On the other hand, speedup of FCLQC linearly increases until 40 threads and increases steadily after that. It is due to some not perfectly optimized parts of FCLQC which are not parallelized, such as merging local statistics. However, it is still convincing that FCLQC shows comparable speedup with highly optimized algorithms such as pigz. We also point out that the overall compression speed of FCLQC is much faster than pigz. The above result justifies that the concurrent implementation using Rust.

In order to evaluate random access decompression speed, we select 30 random quality score line indexes, where 10 of them are small indexes (low), another ten are mid-range indexes (mid), and the last ten are large indexes (high). Then, we measured the average time (seconds) to decompress a quality score line of selected indexes of each range (low, mid, and high). 7-zip and pigz do not support random access, and AQUa fails to random access with some datasets [14]. Therefore, we compare the random access result to LCQS only. Since the thread count of LCQS varies while random access decompression, it is hard to determine the number of threads for FCLQC for a fair comparison. We set FCLQC to use a single thread for a simple comparison, although it can handle multi-threads for random access decompression. Table 6 shows the random access decompression speeds where FCLQC outperforms LCQS except for *syn_read1* and *syn_read2*. This is due to single-thread restriction of FCLQC when the file size is huge. Note that the random access decompression time is nearly half if two threads are allowed for FCLQC. We also note that indexing in FCLQC is not fully optimized; therefore the random access decompression speed depends on the index. Standard deviations (std) of random access decompression times of FCLQC are also provided, where LCQS shows consistent decompression speed (we omit std of LCQS).

**Table 6:** Result of random access decompression speed

Filename	Comparison of random access decompression speed (s)					
	FCLQC			LCQS		
	Low	Mid	High	Low	Mid	High
*SRR554369_1*	$\mathbf{0.148 }\pm \mathbf{0.063 }$	$\mathbf{0.383 }\pm \mathbf{0.075 }$	$\mathbf{0.572 }\pm \mathbf{0.055 }$	50.027	50.082	50.213
*SRR554369_2*	$\mathbf{0.133 }\pm \mathbf{0.052 }$	$\mathbf{0.379 }\pm \mathbf{0.078 }$	$\mathbf{0.567 }\pm \mathbf{0.054 }$	52.310	52.507	52.627
*SRR327342_1*	$\mathbf{0.834 }\pm \mathbf{0.399 }$	$\mathbf{2.194 }\pm \mathbf{0.361 }$	$\mathbf{3.579 }\pm \mathbf{0.371 }$	52.855	55.487	56.429
*SRR327342_2*	$\mathbf{1.073 }\pm \mathbf{0.417 }$	$\mathbf{2.787 }\pm \mathbf{0.440 }$	$\mathbf{4.554 }\pm \mathbf{0.497 }$	54.961	57.307	58.232
*SRR870667_1*	$\mathbf{6.335 }\pm \mathbf{1.534 }$	$\mathbf{14.695 }\pm \mathbf{2.389 }$	$\mathbf{24.424 }\pm \mathbf{2.323 }$	53.417	54.124	52.771
*SRR870667_2*	$\mathbf{6.048 }\pm \mathbf{1.421 }$	$\mathbf{14.175 }\pm \mathbf{2.118 }$	$\mathbf{21.984 }\pm \mathbf{2.465 }$	58.847	58.174	57.997
*syn_read1*	$\mathbf{32.432 }\pm \mathbf{4.864 }$	$73.781 \pm 10.756$	$97.296 \pm 15.459$	60.651	**62.135**	**63.547**
*syn_read2*	$\mathbf{31.541 }\pm \mathbf{4.498 }$	$70.991 \pm 9.648$	$94.623 \pm 13.132$	61.965	**62.456**	**63.165**

Bold denotes the fastest random access decompression

Table 7 shows the results of the decompression speed when the decoder reconstructs the original quality scores sub-files. We set FCLQC to use two threads for *SRR5543692_1* and *SRR5543692_2*, and 16 threads for other datasets. Averaged decompression speeds of FCLQC are 41.36 (MB/s) and 121.28 (MB/s) for two threads and 16 threads, respectively, which outperforms LQCS on all datasets. The peak thread count of LCQS was 120 threads while decompressing, and recall that the number of threads is not an adjustable parameter for LCQS. Although LCQS flexibly varies the thread count while FCLQC is restricted to 16 threads, FCLQC shows better performance (13.9x in *SRR870667_1* and 5.6x in *SRR870667_2*).

**Table 7:** Comparison results of decompression speed

Filename	Decompression speed (MB/s)	
	FCLQC	LCQS
*SRR554369_1*	**40.27**	3.21
*SRR554369_2*	**42.44**	3.07
*SRR327342_1*	**111.66**	11.38
*SRR327342_2*	**116.46**	11.81
*SRR870667_1*	**122.38**	8.21
*SRR870667_2*	**124.95**	18.8
*syn_read1*	**125.85**	10.29
*syn_read2*	**126.40**	10.35

Bold denotes the fastest decompression speed

In Table 8, we also measured the decompression time when the number of threads increases.^2^ Similar to the compression time, the decompression time is (roughly) inversely proportional to the number of threads. Note that the decompression took longer than the compression because of the arithmetic decoder’s binary search.

**Table 8:** Decompression time of FCLQC when the number of threads increases

Filename	Decompression time (s)				
	10	30	60	90	120
*SRR554369_1*	1.49492	0.80012	0.55523	0.51588	0.48690
*SRR554369_2*	1.52766	0.80448	0.56461	0.52217	0.48321
*SRR327342_1*	11.26921	6.51885	4.63464	3.98554	3.35740
*SRR327342_2*	12.21315	7.17441	4.93512	4.40018	4.08363
*SRR870667_1*	67.20655	35.34112	24.08606	21.38290	18.88168
*SRR870667_2*	54.44474	31.43011	21.77509	18.16317	16.11619
*syn_read1*	412.46256	217.53169	151.75177	130.78657	111.23652
*syn_read2*	433.82677	223.34369	153.61791	132.44471	112.75674

### Comparision: compression ratio

The compression ratio is defined by the ratio between the original quality scores file size and the compressed file size. Table 9 shows the compression ratios of compression schemes under the same settings when we measured compression/decompression speeds in Tables 4 and 7. LCQS tends to obtain better compression ratios than the other methods in all datasets. The compression ratios of FCLQC are comparable (or slightly worse) to that of LCQS for most datasets. Compared to AQUa and 7-zip, the proposed algorithm shows better performance, 3% and 4.69% on average, respectively. For all datasets, FCLQC shows a better compression ratio (about 14%) on average than pigz.

**Table 9:** Comparison results of compression ratio

Filename	Compression ratio				
	FCLQC	LCQS	AQUa	7-zip	pigz
*SRR554369_1*	3.02	**3.43**	2.97	2.94	2.59
*SRR554369_2*	3.04	**3.32**	2.93	2.87	2.54
*SRR327342_1*	2.59	**2.79**	2.57	2.51	2.25
*SRR327342_2*	2.42	**2.57**	2.35	2.31	2.09
*SRR870667_1*	2.89	**3.25**	2.86	2.83	2.50
*SRR870667_2*	2.66	**2.86**	2.58	2.54	2.27
*syn_read1*	2.52	**2.62**	2.39	2.29	2.14
*syn_read2*	2.20	**2.32**	2.07	2.07	1.91

Bold denotes the highest compression ratio

**Fig. 3:**
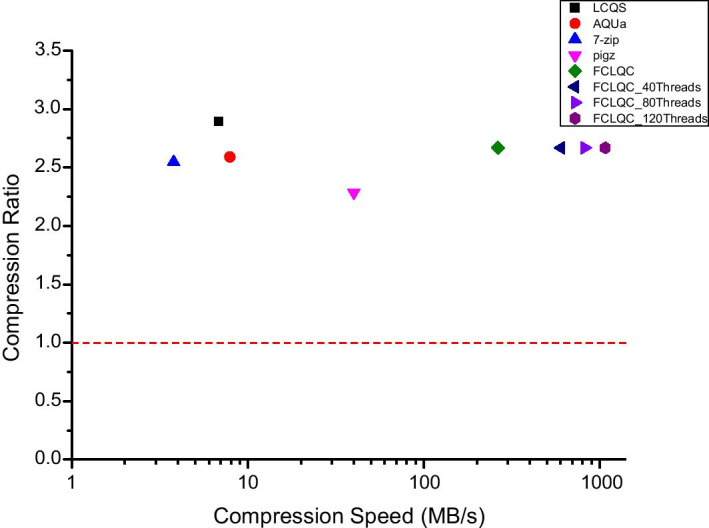
The average compression ratio and the compression of FCLQC and baseline compressors for all dataset

**Fig. 4:**
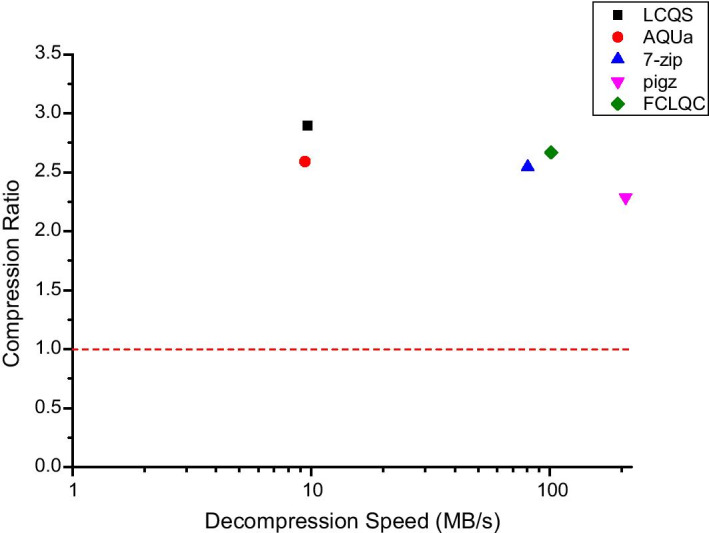
The average compression ratio and decompression speed of FCLQC and baseline compressors for all dataset

Figure 3 visualizes the trade-off between average compression ratio and compression speed of compression schemes for all dataset. Although FCLQC has a lower compression ratio than LCQS, it shows a significantly faster compression speed. Figure 4 shows the average compression ratio and decompression speed of compression schemes for all dataset. In this experiment, LCQS used more than two threads, and all other algorithms used only one thread. Although pigz has a faster decompression speed than other algorithms, it does not support random access and the compression rate is degraded up to 25% compared to LCQS. The performance degradation of FCLQC is not significant considering the extreme boosts on compression/decompression speeds and the generality of the algorithm.

It is clear that the better compression ratio is one of the most important goals for compression algorithms; however, it is not the only criterion. There are applications where the compression speed is a bottleneck, many commercial compression algorithms provide an option to sacrifice compression rate to achieve better compression speed. For example, pigz has a “best” option, which provides the best compression ratio but slow. On the other hand, pigz also has a “fast” option with faster compression, but the compression ratio is worse. More precisely, if we compress *SRR_554369_1* using pigz, the compression ratio is 2.59 with the “best” option and 2.23 with the “fast” option. We believe that FCLQC is a reasonable choice for speed sensitive applications.

## Conclusions

We proposed a new lossless quality scores compressor FCLQC, which focuses on the algorithm’s running time. We implemented FCLQC using Rust and achieved thread safety via concurrent programming. FCLQC was evaluated on various quality score datasets and showed significant boosts on compression speed while maintaining the compression ratio. Also, FCLQC is universal since it does not have any assumptions on sequencing technologies and supports desired features such as random access. Thus, FCLQC is a good candidate for FASTQ file compression in practice, where the compression and decompression speed is a bottleneck.

### Availability and requirements

- Project name: FCLQC.
- Project home page: https://github.com/Minhyeok01/FCLQC.
- Operating systems: Linux/Windows.
- Programming language: Rust.
- Other requirements: cargo 1.42.0 or higher.
- License: The MIT License.
- Any restrictions to use by non-academics: For commercial use, please contact the authors.

## Data Availability

The synthetic datasets are available at SMaSH http://smash.cs.berkeley.edu/datasets.html (Identifiers: Synthetic Datasets/Venter) and the others are available at NCBI https://www.ncbi.nlm.nih.gov/ (Identifiers: SRR554369, SRR327342, and SRR870667). The implementation of FCLQC can be downloaded from https://github.com/Minhyeok01/FCLQC.

## Abbreviations

GB: Gigabyte
HGP: Human genome project
IO: Input and output
LTS: Long term support
MB: Megabyte
MPEG-G: Moving Picture Experts Group—Genomic information
NGS: Next generation sequencing
SMaSH: Synthetic, mouse and sampled human data

## Footnotes

1 Note that preprocessing (mostly splitting) took 26 s when we split the 20 GB quality scores file into 120 sub-files using Linux command. It is negligible compared to other parts of compression.

2 To focus on the role of the number of threads, we ignore the post-processing (merging sub-files) while measuring decompression time. Note that it took 10 s to merge all decompressed sub-files for the reconstructed quality score file of size 20 GB. This is relatively small compared to the total decompression time.

## References

[1] Mardis ER (2011). A decade’s perspective on DNA sequencing technology. Nature.

[2] Stephens ZD, Lee SY, Faghri F, Campbell RH, Zhai C, Efron MJ, Iyer R, Schatz MC, Sinha S, Robinson GE (2015). Big data: astronomical or genomical?. PLoS Biol.

[3] Chandak S, Tatwawadi K, Ochoa I, Hernaez M, Weissman T (2019). Spring: a next-generation compressor for FASTQ data. Bioinformatics.

[4] Al Yami S, Huang C-H (2019). LFastqC: a lossless non-reference-based FASTQ compressor. PLoS ONE.

[5] Deorowicz S (2020). FQSqueezer: k-mer-based compression of sequencing data. Sci Rep.

[6] Bonfield JK, Mahoney MV (2013). Compression of FASTQ and SAM format sequencing data. PLoS ONE.

[7] Hernaez M, Ochoa I, Weissman T. A cluster-based approach to compression of quality scores. In: 2016 data compression conference (DCC). IEEE; 2016. p. 261–70. 10.1109/DCC.2016.49 PMC5649045 29057318

[8] Malysa G, Hernaez M, Ochoa I, Rao M, Ganesan K, Weissman T (2015). QVZ: lossy compression of quality values. Bioinformatics.

[9] Bonfield JK, McCarthy SA, Durbin R (2019). Crumble: reference free lossy compression of sequence quality values. Bioinformatics.

[10] Voges J, Hernaez M, Mattavelli M, Ostermann J. An introduction to MPEG-G: The first open ISO/IEC standard for the compression and exchange of genomic sequencing data. In: Proceedings of the IEEE; 2021.

[11] Nicolae M, Pathak S, Rajasekaran S (2015). LFQC: a lossless compression algorithm for FASTQ files. Bioinformatics.

[12] Cochrane G, Cook CE, Birney E (2012). The future of DNA sequence archiving. GigaScience.

[13] Paridaens T, Van Wallendael G, De Neve W, Lambert P (2018). AQUA: an adaptive framework for compression of sequencing quality scores with random access functionality. Bioinformatics.

[14] Fu J, Ke B, Dong S (2020). LCQS: an efficient lossless compression tool of quality scores with random access functionality. BMC Bioinform.

[15] Klabnik S, Nichols C. The rust programming language; 2018. https://doc.rust-lang.org/book/ch16-00-concurrency.html.

[16] Research M. Rust; 2010. https://www.rust-lang.org/.

[17] Fulton KR, Chan A, Votipka D, Hicks M, Mazurek ML. Benefits and drawbacks of adopting a secure programming language: rust as a case study. In: Seventeenth symposium on usable privacy and security ($$\{$$SOUPS$$\}$$ 2021); 2021. p. 597–616.

[18] Perkel JM (2020). Why scientists are turning to rust. Nature.

[19] Stack Overflow Developer Survey 2020. https://insights.stackoverflow.com/survey/2020.

[20] Köster J (2016). Rust-bio: a fast and safe bioinformatics library. Bioinformatics.

[21] Metzker ML (2010). Sequencing technologies—the next generation. Nat Rev Genet.

[22] Voelkerding KV, Dames SA, Durtschi JD (2009). Next-generation sequencing: from basic research to diagnostics. Clin Chem.

[23] Haque F, Li J, Wu H-C, Liang X-J, Guo P (2013). Solid-state and biological nanopore for real-time sensing of single chemical and sequencing of DNA. Nano Today.

[24] McCarthy A (2010). Third generation DNA sequencing: pacific biosciences’ single molecule real time technology. Chem Biol.

[25] Rusk N (2011). Torrents of sequence. Nat Methods.

[26] Massingham T. simNGS—software for simulating next generation sequencing data; 2012. https://www.ebi.ac.uk/goldman-srv/simNGS/.

[27] Talwalkar A, Liptrap J, Newcomb J, Hartl C, Terhorst J, Curtis K, Bresler M, Song YS, Jordan MI, Patterson D (2014). SM a SH: a benchmarking toolkit for human genome variant calling. Bioinformatics.

